# Formulation and Characterization of Drug Loaded Nonionic Surfactant Vesicles (Niosomes) for Oral Bioavailability Enhancement

**DOI:** 10.1155/2014/959741

**Published:** 2014-02-02

**Authors:** Sunil Kamboj, Vipin Saini, Suman Bala

**Affiliations:** M.M. College of Pharmacy, Maharishi Markandeshwar University, Mullana, Ambala 133207, Haryana, India

## Abstract

Nonionic surfactant vesicles (niosomes) were formulated with an aim of enhancing the oral bioavailability of tenofovir disoproxil fumarate (TDF), an anti-HIV drug. Niosomes were formulated by conventional thin film hydration technique with different molar ratios of surfactant, cholesterol, and dicetyl phosphate. The formulated niosomes were found spherical in shape, ranging from 2.95 **μ**m to 10.91 **μ**m in size. Vesicles with 1 : 1 : 0.1 ratios of surfactant : cholesterol : dicetyl phosphate with each grade of span were found to have higher entrapment efficiencies, which were further selected for *in vitro* and *in vivo* studies. Vesicles formulated with sorbitan monostearate were found to have maximum drug release (99.091%) at the end of 24 hours and followed zero order release kinetics. The results of *in vivo* study revealed that the niosomes significantly enhanced the oral bioavailability of TDF in rats after a dose of 95 mg/kg. The average relative bioavailability of niosomes in relation to plane drug solution was found to be 2.58, indicating more than twofold increase in oral bioavailability of TDF. Significant increase in mean residential time (MRT) was also found, reflecting release retarding efficacy of the vesicles. In conclusion, niosomes could be a promising delivery for TDF with improved oral bioavailability and prolonged release profiles.

## 1. Introduction

Tenofovir disoproxil fumarate (TDF) is antiretroviral drug, acting by blocking the enzyme reverse transcriptase, which is crucial to viral production in HIV-infected people. It has been ascertained a drug of choice in the treatment of HIV-1 infection and hepatitis-B in humans either alone or in combination with other drug molecules [1, 2]. The major problem with the therapy of TDF is its poor bioavailability (25%), which may be due to its poor log⁡⁡*P* and poor permeability across the biological membrane of gastrointestinal tract [3].

Several novel approaches, like prodrugs, microemulsions, liposomes, niosomes, bilosomes, and so forth, have been used by the researchers for improving the gastric absorption of the compounds, which have low permeability [4]. Out of all the approaches, vesicles were found to have the distinct advantages over other approaches because these vesicles act as drug reservoirs and the rate of drug release can be controlled by modification of their compositions [5]. Although liposomes have been studied as an effective vesicular drug delivery system for oral as well as transdermal routes for improving the absorption of the drugs, niosomes are preferred over liposomes due to their higher chemical stability, economy, and simple practical methods of preparation without the use of pharmaceutically unaccepted solvents [6]. Niosomes are the surfactant vesicles which have been prepared from different nonionic surfactants. These are spherical lipid bilayers capable of entrapping water soluble molecules within an aqueous domain or alternatively lipid molecules within lipid bilayers. They may be unilamellar or multilamellar depending upon the method used for their preparation. In recent years, niosomes have been extensively studied for the potential to serve as carriers for delivery of drugs, antigens, hormones, and other bioactive agents for an extended period of time or to a specific organ of the body [7, 8]. In the present study, TDF loaded niosomes were prepared and evaluated for their* in vitro *and* in vivo* characteristics in an attempt to improve the oral bioavailability of the drug and also to extend the drug release for prolonged period of time.

## 2. Materials and Methods

### 2.1. Materials

Tenofovir disoproxil fumarate (TDF) was supplied as a gift sample from Micro Labs, Bangalore. Cholesterol (CHOL), sorbitan laurate (span 20), sorbitan monopalmitate (span 40), sorbitan monostearate (span 60), sorbitan oleate (span 80), chloroform, and sodium hydroxide were purchased from Qualichem Specialties Pvt. Ltd., Mumbai. Dicetyl phosphate (DCP) was purchased from Sigma Aldrich, Bangalore. All other chemicals were of analytical grade and procured from the authentic sources. Dialysis membrane (30/15 mm flat width/diameter) was purchased from Himedia, Mumbai.

### 2.2. Formulation of Drug Loaded Niosomes

Drug loaded niosomes were formulated by conventional thin film hydration technique. Different grades of span such as sorbitan laurate, sorbitan monopalmitate, sorbitan monostearate, and sorbitan oleate in different molar ratios of surfactant : CHOL : DCP as 2.5 : 1 : 0.1, 2 : 1 : 0.1, 1.5 : 1 : 0.1, 1 : 1 : 0.1, and 1 : 1.5 : 0.1 (total weight of lipid mixture kept constant as 100 mg) were used. Lipid mixture : drug ratio was kept 1 : 0.5 in all the batches. Accurately weighed quantities of surfactant, CHOL, and DCP were dissolved in 10 mL chloroform using a 100 mL round bottom flask. The lipid solution was evaporated by rotary flash evaporator (Perfit, India) under reduced pressure at a temperature of 60 ± 2°C. The flask was rotated at 120 rpm until a smooth and dry lipid film was obtained. The film was hydrated with 10 mL phosphate buffer saline (PBS) of pH 7.4 containing drug for 3 hours at 60 ± 2°C with gentle shaking. The niosomal suspension was further stabilized by keeping at 2–8°C for 24 hours.

### 2.3. Visual Observation

All the prepared batches were visually observed for turbidity and flocculation in transparent containers. The selected batches (with good entrapment efficiency) were also observed for sedimentation. The experiment was performed in triplicate and results for sedimentation rate were reported on the bases of bottom view of the containers having niosomal preparations (Table 1).

### 2.4. Vesicle Size Measurement

The average vesicle size of the prepared niosomes was measured by using optical microscope (Vaiseshika 7001-IMS) and the vesicle size distribution studies were performed on the optimized batches by measuring the size of randomly selected 100 niosomes vesicles from each formulation. The vesicles were also studied by scanning electron microscopy (SEM) technique to check their shape at higher magnification values.

### 2.5. Zeta Potential Measurement

The surface charge of the vesicles plays an important role in the *in vivo* performance of niosomes. The significance of zeta potential is that its value can be related to the stability of vesicular formulations. The zeta potential indicates the degree of repulsion between adjacent, similarly charged particles in dispersion system. Zeta potential of suitably diluted niosomal dispersion was determined using Zetasizer Nano ZS-90 (Malvern Instruments Ltd., UK) at 25°C. The working principle of the instrument is electrophoretic light scattering (ELS), which determines electrophoretic movement of charged particles under an applied electric field from doppler shift of scattered light, for zeta potential determination [9].

### 2.6. Entrapment Efficiency

Entrapment efficiency of drug loaded niosomes was determined after separation of unentrapped drug, which was performed by cooling centrifugation (Remi, C-24DL) at 12,000 rpm for 30 min at 4°C. The supernatant liquid was collected separately. The separated vesicles were washed with PBS and the washings were mixed with supernatant liquid. The vesicles were suspended in 3 mL PBS and placed in a dialysis bag. The dialysis bag after tying at both ends was immersed in 200 mL PBS, maintained at 37°C, and stirred overnight by using magnetic stirrer [10]. Drug was estimated spectrophotometrically at *λ*_max⁡_ of 261 nm, against PBS as blank. The percentage of entrapped drug was calculated by applying the following equation:

(1) $$\begin{array}{c} \%\;\text{Entrapment}=\frac{\left(D_{E}\times 100\right)}{\left(D_{I}\right)}, \end{array}$$

where *D*_*E*_ is the amount of entrapped drug and *D*_*I*_ is the initial amount of drug.

The entrapped drug was also verified by estimating the unentrapped drug in the supernatant liquid separated in the initial step [11].

### 2.7. *In Vitro* Drug Release Study

*In vitro* release pattern of niosomal suspension was carried out according to the method reported by Attia et al. [12]. The formulations with each grade of surfactant, which showed the better entrapment efficiencies (F4, F9, F14, and F19), were further selected for carrying out *in vitro* release studies. Buffers of different pH were used for *in vitro* drug release studies to simulate stomach and blood pH and also to evaluate the effect of pH on drug release. Dialysis tube containing the measured amount of drug loaded niosomal dispersion was initially placed in magnetically stirred 200 mL of 0.1 N HCL at 37 ± 5°C, and then after the completion of 2 hours of the study, the test media were replaced with PBS pH 7.4 and the test was continued for a total period of 24 hours. Aliquots of 5 mL samples of dialysate were withdrawn periodically at 0.5, 1, 2, 3, 4, 6, 8, 10, 12, 18, and 24 hours and immediately replenished with the same volume of buffer medium. The drug content was determined spectrophotometrically at *λ*_max⁡_ of 261 nm. Results are the mean of three runs.

### 2.8. Release Kinetics Modeling

For the characterization of the release kinetics studies and to determine the release mechanism of drug, the results of *in vitro* studies were fitted with several kinetics models as follows.

Zero order rate equation:

(2) $$\begin{array}{c} Q_{t}=Q_{0}+K_{0}t, \end{array}$$

where *Q*_*t*_ is the amount of drug dissolved in time *t*, *Q*_0_ is initial amount of drug in solution, and *K*_0_ is zero order release constant.

First order rate equation:

(3) $$\begin{array}{c} \log C=\log C_{0}-\frac{K_{t}}{2.303}, \end{array}$$

where *C*_0_ is the initial concentration of drug, *K* is first order release constant, and *t* is time.

Higuchi's model:

(4) $$\begin{array}{c} Q=K_{H}t^{1/2}, \end{array}$$

where *Q* is the amount of drug released in time *t* per unit area, *K*_*H*_ is Higuchi dissolution constant.

Hixson-Crowell model:

(5) $$\begin{array}{c} W_{0}^{1/3}-W_{t}^{1/3}=\kappa t, \end{array}$$

where *W*_0_ is the initial amount of drug in the niosomes, *W*_*t*_ is the remaining amount of drug in the niosomes at time *t*, and *κ* (kappa) is a constant incorporating the surface-volume relation.

To find out the mechanism of drug release, the *in vitro* release data of all niosomal formulations were fitted into Korsmeyer and Peppas equation [13]:

(6) $$\begin{array}{c} \frac{M_{t}}{M_{\infty }}=Kt^{n}, \end{array}$$

where *M*_*t*_/*M*_*∞*_ is a fraction of drug release at time *t*, *K* is the release rate constant, and *n* is the release exponent. The value of exponent (*n*) indicates the mechanism of drug release.

### 2.9. *In Vivo* Study

*In vivo* study was performed according to the method reported by Jadonetal. [14] and in accordance with the protocol approved by the Institutional Animal Ethical Committee of M.M. College of Pharmacy, M.M. University, Mullana (protocol no. MMCP/IAEC/11/18).

Eighteen male albino rats (Sprague-Dawely strain) weighing 145 to 155 g were selected for study. The animals were divided in to three groups, each group containing six animals. The animals were fasted overnight for 12 hours. On the study day, the first group was fed with PBS of pH 7.4 orally. Second and third groups were treated with plain TDF solution in PBS and TDF loaded niosomal formulation (equivalent to 95 mg TDF per kilogram of body weight), respectively, by oral route. Blood samples were withdrawn from retro-orbital plexus of eye at 0.5, 1, 1.5, 2, 4, 6, 8, 12, and 24 hours after dosing, in eppendorf tubes containing 1-2 drops of 10% EDTA solution. The blood samples were centrifuged by cooling centrifuge at 2000 rpm for 10 min. The temperature was maintained 4°C during the centrifugation. The separated plasma samples were analyzed by double beam UV-visible spectrophotometer at 261 nm.

## 3. Results and Discussion

### 3.1. Visual Observation

All the prepared batches were visually observed for turbidity and flocculation in transparent containers and were found to be turbid and whitish in colour. However, the selected batches (with good entrapment efficiencies) were also evaluated for sedimentation while keeping at 4°C for 3 months in the transparent containers. The results revealed that in all the batches, except F14, sedimentation started after 30 days of the storage but the niosomal formulation, formulated with sorbitan monostearate, was found in good dispersible form indicating the good physical stability (Table 2).

### 3.2. Vesicle Size Measurement

**Figure 1:**
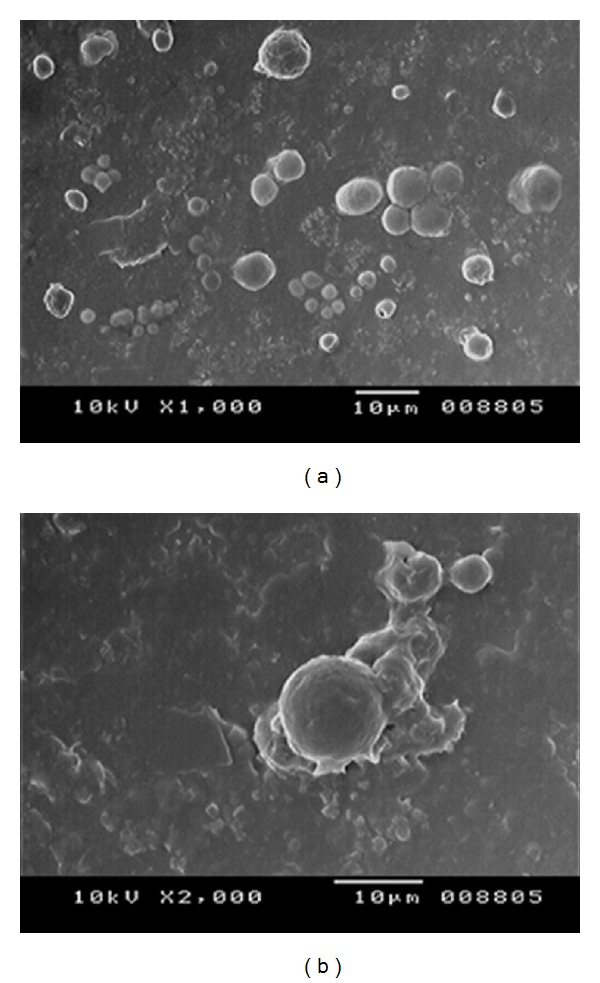
(a) SEM image of TDF loaded niosomes, (b) spherical structure of niosome at higher magnification.

**Figure 2:**
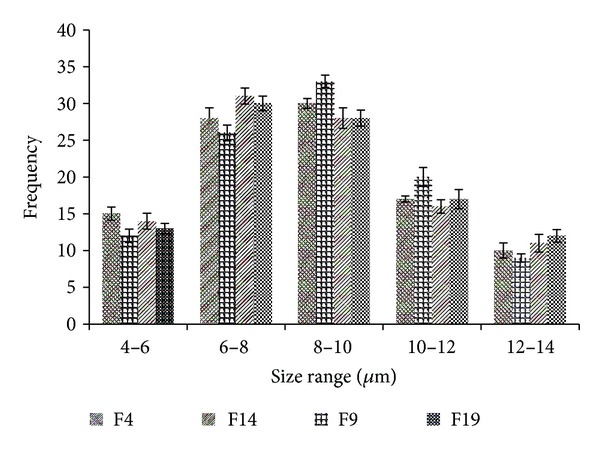
Size distribution plots of all optimized batches.

The formulated niosomal vesicles were found to be spherical in shape (Figure 1), ranging from 2.95 *μ*m to 10.91 *μ*m in size. The details of vesicles size ranges for all the batches and size distribution plots for the optimized batches are given in Table 3 and Figure 2, respectively. The effect of surfactant HLB value on vesicles size has come forward; as the HLB value of the surfactants moves towards the hydrophilicity, the vesicles size was found to be increasing. Niosomal vesicles formulated with sorbitan laurate (HLB 8.6) got higher size range than the vesicles formulated with sorbitan oleate (HLB 4.3). Further the vesicles formulated with sorbitan monostearate (HLB 4.7) were found to have almost similar size range as with sorbitan oleate, which may be due to the adjacent HLB range of these surfactants. The effect of HLB values of the surfactants on the vesicles size could be explained as the surface energy increases with increasing the hydrophilicity; also the water uptake of the surfactants increases with the HLB values moving towards hydrophilic region and both reasons result in larger size of vesicles [15, 16].

Further it has also been found that, with increasing the CHOL concentration, vesicle size also increases. CHOL is the main additive which affects the physical stability of the vesicles as it provides the rigidity to the bilayer membrane. It strengthens the bilayer and diminishes the bilayer fluidity by eliminating the phase transition temperature peak of the vesicles [15, 17].

### 3.3. Zeta Potential Measurement

The values of zeta potential for all the batches are illustrated in Table 3, which were found in range of −16 to −91. The results revealed that the zeta values of the vesicles increase towards negative with increasing the HLB values of the surfactants. The niosomal vesicles formulated with sorbitan oleate were found to have the least zeta values, ranging from −16 to −45 compared to the vesicles formulated with sorbitan laurate, showing higher zeta values ranging from −25 to −91. The effect of HLB values of surfactants on zeta potential could be explained in terms of surface energy, which tends to increase with increase in HLB values towards the hydrophilicity. Increase in surface energy of the vesicles leads to increase the values of zeta potential towards negative [18, 19].

### 3.4. Entrapment Efficiency

**Figure 3:**
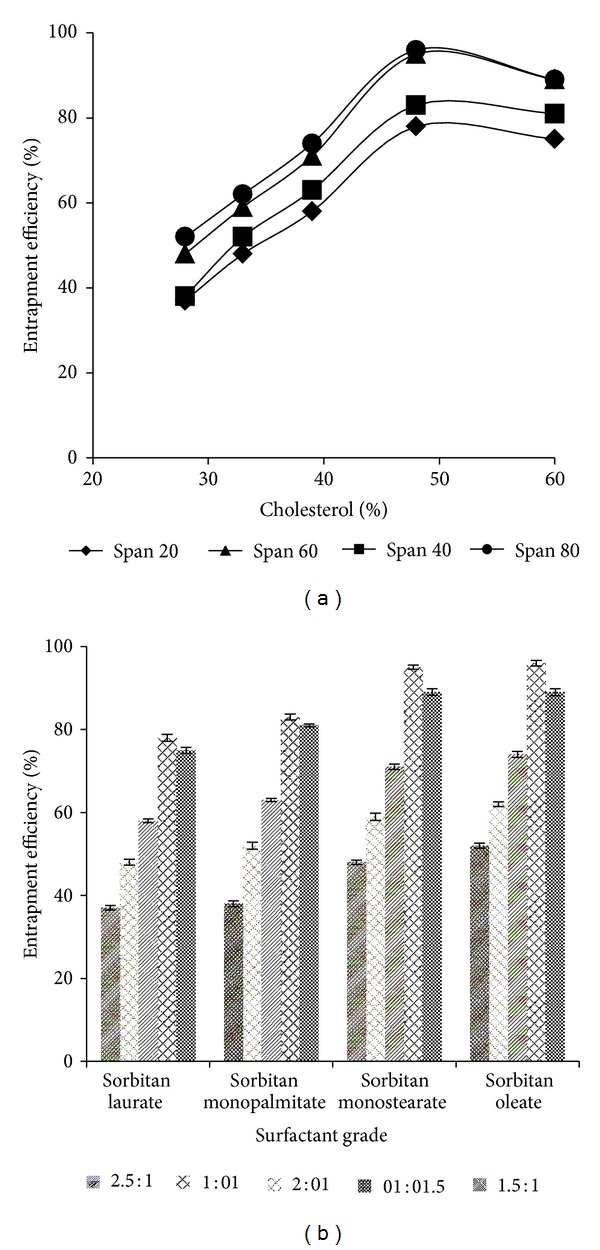
(a) % entrapment efficiency versus % CHOL indicating the effect of CHOL concentration on % entrapment efficiency, (b) % entrapment efficiency versus surfactant grades with several surfactant : CHOL ratios.

The entrapment efficiency is an important parameter for the characterization of niosomal vesicles which was found ranging from 37% to 96% and tabulated in Table 3. The results revealed the effect of lipid concentration and type of surfactant used on percentage entrapment efficiency of drug in niosomal vesicles. The percentage entrapment efficiency increased with increasing the CHOL concentration at a ratio up to 1 : 1, after that it became constant (Figure 3). The incorporation of CHOL leads to increase the viscosity of the formulation, indicating more membrane rigidity and good physical stability [10]. The percentage of drug entrapment was found to be good with surfactant : CHOL ratio 1 : 1 with all grades of surfactants but sorbitan monostearate showed the highest drug entrapment compared to other grades (Figure 3).

### 3.5. *In Vitro* Drug Release Study

**Figure 4:**
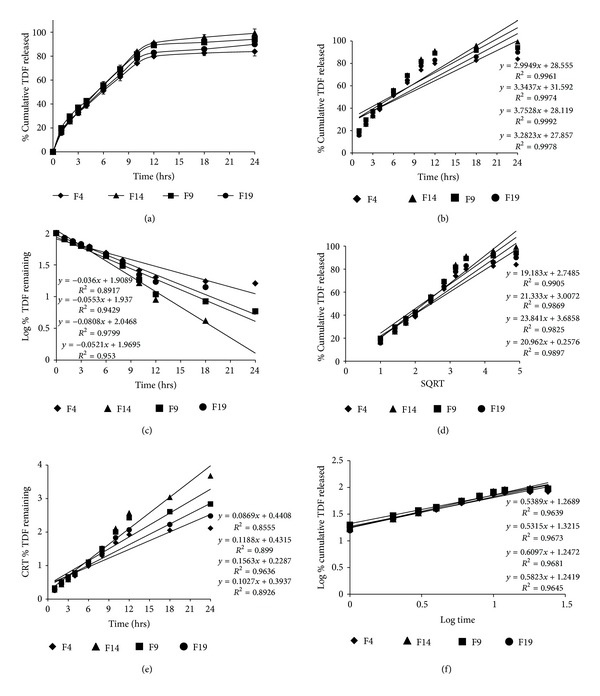
Comparative plots of (a) *in vitro* release profile, (b) zero order release kinetics, (c) first order release kinetics, (d) Higuchi (SQRT) release kinetics, (e) Hixson-Crowell model, and (f) Korsmeyer-Peppas model for the selected niosomal formulations.

*In vitro* release studies were performed by dialysis method. The release profile of TDF is given in Figure 4(a), shown to be retarded for 24 hours. The percentage of TDF released at the end of 24 hours is given in Table 3. The results revealed that the maximum percentage of TDF (99.091%) was released from F14 (with sorbitan monostearate), followed by F9, that is, 94.128% (with sorbitan monopalmitate), F19, that is, 89.901% (with sorbitan oleate), and F4, that is, 83.866% (with sorbitan laurate) at the end of 24 hours.

### 3.6. Release Kinetics Modeling

The *in vitro* release data was fitted to various release kinetics models to predict the release mechanism of drug from the niosomes. The results revealed that all the formulations were best explained by zero order release (plots show highest linearity) followed by Higuchi release kinetics indicating that the concentration was independent of drug release. But the formulation F14 showed the highest linearity among all the formulations indicating the best zero order release kinetics (Figure 4). However, the drug release was also found to be very close to Higuchi kinetics in all the formulations, explaining that the drug diffuses at a slower rate as the distance for diffusion increases, referred to the square root kinetics (Table 4).

The Korsmeyer-Peppas model indicated a good linearity for all the niosomal formulations and the values of release exponent (*n*) were found in the range of 0.45 to 0.89 [13], which indicate a coupling of diffusion and erosion mechanism so-called anomalous release mechanism. This may indicate that the drug release was controlled by more than one process.

### 3.7. *In Vivo* Study

**Figure 5:**
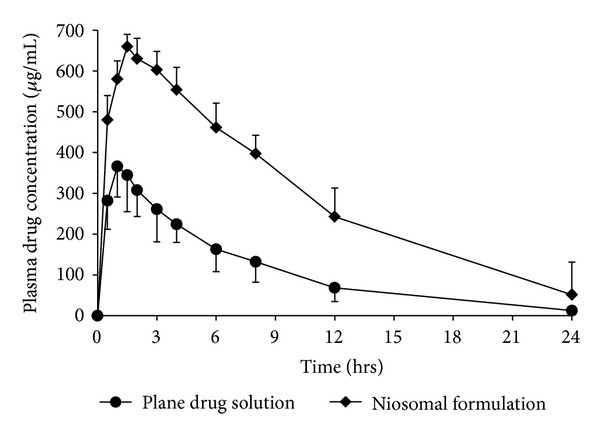
Mean plasma TDF concentration time profiles (±SD) in rats after oral administration of plane drug solution (●) and niosomal formulation (♦) as a single dose of 95 mg/kg (*n* = 6).

The average plasma drug concentration time profile in rats after a single oral dose of TDF (95 mg/kg) as plane drug solution and niosomal dispersion (containing both entrapped and unentrapped drug) is shown in Figure 5. Various pharmacokinetics parameters of TDF were calculated from individual profiles, the mean values of which are given in Table 5. The niosomal formulation showed significantly (*P* < 0.05) higher values for AUC_0→*∞*_, *C*_max⁡_, *T*_max⁡_, *T*_1/2_, and MRT and significantly (*P* < 0.05) lower values for absorption (*K*_*a*_) and elimination (*K*) rate constants as compared with plane TDF solution. The increase in AUC_0→*∞*_ and MRT values and decrease in *K*_*a*_ value reflect the release retarding effect of niosomal formulation [12], which was also investigated by *in vitro* release studies in terms of controlled release.

The higher values of AUC_0→*∞*_ and *C*_max⁡_ for niosomal formulation may be due to the enhanced absorption of drug loaded niosomes through gastrointestinal track after oral administration [20] and *T*_max⁡_, *T*_1/2_, and MRT may be due to the release retarding effect of niosomal vesicles. Further, the AUC_0→*∞*_ value for niosomal formulation was compared with that of plane drug solution to determine the relative bioavailability and the mean ratio was found to be 2.58 (±0.98), showing more than twofold increase in the oral bioavailability of TDF by niosomal formulation.

## 4. Conclusion

All the formulated niosomal vesicles were found to be spherical in shape ranging from 2.95 *μ*m to 10.91 *μ*m in size and have zeta values within range. The percentage of drug entrapment was found to be higher with surfactant : CHOL ratio 1 : 1 with all grades of surfactants but sorbitan monostearate showed the highest drug entrapment compared to other grades. *In vitro* study revealed that formulation F14 (with sorbitan monostearate) showed maximum 99.091% drug release at the end of 24 hours. Further, the *in vitro* release profile was fitted to various release kinetics models to predict the release mechanism of drug from the niosomes and the results revealed that all the formulations were best explained by zero order release. The results of *in vivo* study revealed more than two fold increase in oral bioavailability of TDF by niosomal vesicles compared with the plane TDF solution in same dose. So the prepared TDF loaded niosomal vesicles could be the promising drug delivery system for controlled release of TDF.

## Figures and Tables

**Table 1:** Bottom view of container showed different degrees of sedimentation.

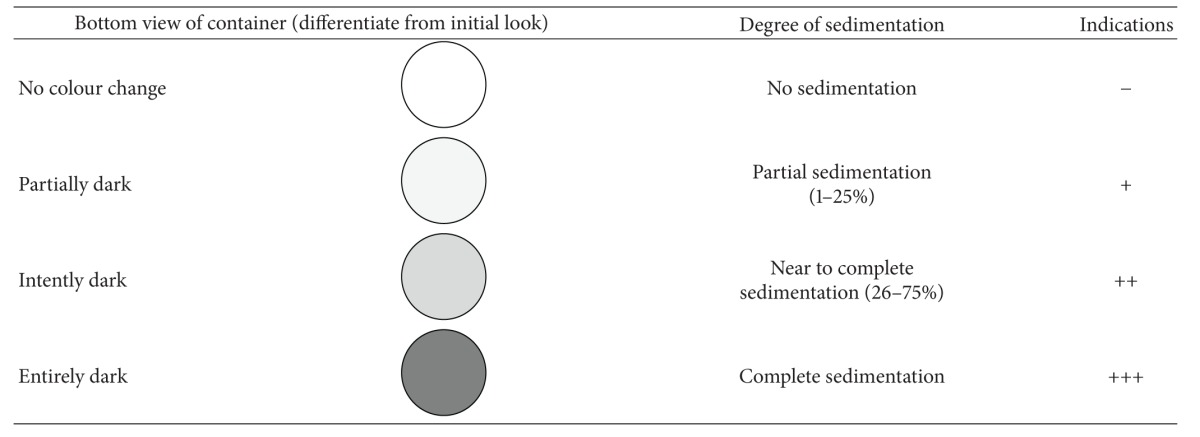

**Table 2:** Degree of sedimentation of TDF loaded niosomes after being stored at 4°C for 3 months.

Formulation code	0 day	15 days	30 days	45 days	60 days	90 days
F4	−	−	+	+	++	+++
F9	−	−	+	+	++	++
F14	−	−	−	−	+	+
F19	−	−	+	+	++	++

**Table 3:** Composition and characterization of niosomal formulations.

Formulation code	Surfactant grade	Surfactant : CHOL : DCP ratio	Mean vesicle diameter (*μ*m)*	Zeta potential (mV)*	Entrapment efficiency (%)*	% Cumulative drug released (at the end of 24 hours)*
F1	Sorbitan laurate	2.5 : 1 : 0.1	7.12 ± 0.75	−91 ± 0.19	37 ± 0.56	—
F2		2 : 1 : 0.1	8.01 ± 0.61	−87 ± 0.13	48 ± 0.69	—
F3		1.5 : 1 : 0.1	8.70 ± 1.24	−56 ± 0.23	58 ± 0.42	—
F4		1 : 1 : 0.1	9.03 ± 0.76	−29 ± 0.12	78 ± 1.08	83.86 ± 3.72
F5		1 : 1.5 : 0.1	10.91 ± 0.86	−25 ± 0.15	75 ± 0.73	—

F6	Sorbitan monopalmitate	2.5 : 1 : 0.1	5.70 ± 0.75	−61 ± 0.16	38 ± 0.71	—
F7		2 : 1 : 0.1	7.84 ± 0.96	−52 ± 0.21	52 ± 0.82	—
F8		1.5 : 1 : 0.1	8.65 ± 0.81	−48 ± 0.24	63 ± 0.36	—
F9		1 : 1 : 0.1	8.90 ± 1.30	−26 ± 0.18	83 ± 0.71	94.12 ± 4.82
F10		1 : 1.5 : 0.1	9.85 ± 0.78	−22 ± 0.17	81 ± 0.37	—

F11	Sorbitan monostearate	2.5 : 1 : 0.1	3.98 ± 0.34	−46 ± 0.15	48 ± 0.52	—
F12		2 : 1 : 0.1	4.31 ± 0.67	−39 ± 0.17	59 ± 1.01	—
F13		1.5 : 1 : 0.1	4.93 ± 1.16	−28 ± 0.23	71 ± 0.65	—
F14		1 : 1 : 0.1	6.82 ± 0.76	−23 ± 0.21	95 ± 0.51	99.09 ± 4.78
F15		1 : 1.5 : 0.1	8.55 ± 0.86	−22 ± 0.18	89 ± 0.79	—

F16	Sorbitan oleate	2.5 : 1 : 0.1	2.95 ± 0.87	−45 ± 0.19	52 ± 0.63	—
F17		2 : 1 : 0.1	4.17 ± 0.86	−37 ± 0.20	62 ± 0.54	—
F18		1.5 : 1 : 0.1	4.59 ± 0.56	−25 ± 0.21	74 ± 0.74	—
F19		1 : 1 : 0.1	6.31 ± 0.75	−21 ± 0.11	96 ± 0.66	89.90 ± 3.99
F20		1 : 1.5 : 0.1	8.42 ± 0.65	−16 ± 0.14	89 ± 0.82	—

*The data were reported as an average of 3 measurements (mean ± S.D.).

**Table 4:** Release kinetics of niosomal formulations.

Formulation code	Zero order		First order		Higuchi		Hixson-Crowell model		Korsmeyer-Peppas model	
	*r* ^2^	*K*_0_ (h^−1^)	*r* ^2^	*K*_1_ (h^−1^)	*r* ^2^	*K*_*H*_	*r* ^2^	*K*_HC_ (h^−1/3^)	*r* ^2^	*n*
F4	0.996	2.994	0.891	0.082	0.990	19.183	0.855	0.086	0.963	0.54
F9	0.997	3.343	0.942	0.126	0.986	21.333	0.899	0.118	0.967	0.53
F14	0.999	3.752	0.979	0.184	0.982	23.841	0.963	0.156	0.968	0.61
F19	0.997	3.282	0.953	0.119	0.989	20.962	0.892	0.102	0.964	0.58

**Table 5:** Pharmacokinetic parameters of TDF in rats after oral administration of a single dose of 95 mg/kg as plane drug solution and niosomal formulation.

Parameters	Plane drug solution*	Niosomal formulation*
AUC_0→*∞*_ (*μ*g·h·mL^−1^)	2781.51 ± 622	7184.9 ± 851
*C*_max⁡_ (*μ*g·mL^−1^)	365.91 ± 98	659.9 ± 104
*T*_max⁡_ (h)	1.20 ± 0.81	2.51 ± 0.17
*T*_1/2_ (h)	6.32 ± 1.02	11.81 ± 3.42
MRT (h)	9.09 ± 3.12	17.03 ± 5.4
*K*_*a*_ (h^−1^)	1.08 ± 0.27	0.86 ± 0.12
*K* (h^−1^)	0.19 ± 0.09	0.07 ± 0.04

*The data were reported as an average of 6 measurements (mean ± S.D.).
